# Oceanographic Variability induced by Tides, the Intraseasonal Cycle and Warm Subsurface Water intrusions in Maxwell Bay, King George Island (West-Antarctica)

**DOI:** 10.1038/s41598-019-54875-8

**Published:** 2019-12-09

**Authors:** P. J. Llanillo, C. M. Aiken, R. R. Cordero, A. Damiani, E. Sepúlveda, B. Fernández-Gómez

**Affiliations:** 1 Departamento de Física, Facultad de Ciencia, Universidad de Santiago de Chile, Santiago, Chile; 2 Estación Costera de Investigaciones Marinas, Facultad de Ciencias Biológicas, Pontificia Universidad Católica, Santiago, Chile; 3 Departamento de Ingeniería Hidráulica y Ambiental, Facultad de Ingeniería, Pontificia Universidad Católica, Santiago, Chile; 4 Center for Environmental Remote Sensing, Chiba University, Chiba, Japan; 5 Universidad de Chile, Santiago, Chile

**Keywords:** Physical oceanography, Physical oceanography

## Abstract

We examine the hydrographic variability induced by tides, winds, and the advance of the austral summer, in Maxwell Bay and tributary fjords, based on two recent oceanographic campaigns. We provide the first description in this area of the intrusion of relatively warm subsurface waters, which have led elsewhere in Antarctica to ice-shelf disintegration and tidewater glacier retreat. During flood tide, meltwater was found to accumulate toward the head of Maxwell Bay, freshening and warming the upper 70 m. Below 70 m, the flood tide enhances the intrusion and mixing of relatively warm modified Upper Circumpolar Deep Water (m-UCDW). Tidal stirring progressively erodes the remnants of Winter Waters found at the bottom of Marian Cove. There is a buoyancy gain through warming and freshening as the summer advances. In Maxwell Bay, the upper 105 m were 0.79 °C warmer and 0.039 PSU fresher in February than in December, changes that cannot be explained by tidal or wind-driven processes. The episodic intrusion of m-UCDW into Maxwell Bay leads to interleaving and eventually to warming, salinification and deoxygenation between 80 and 200 m, with important implications for biological productivity and for the mass balance of tidewater glaciers in the area.

## Introduction

**Figure 1:**
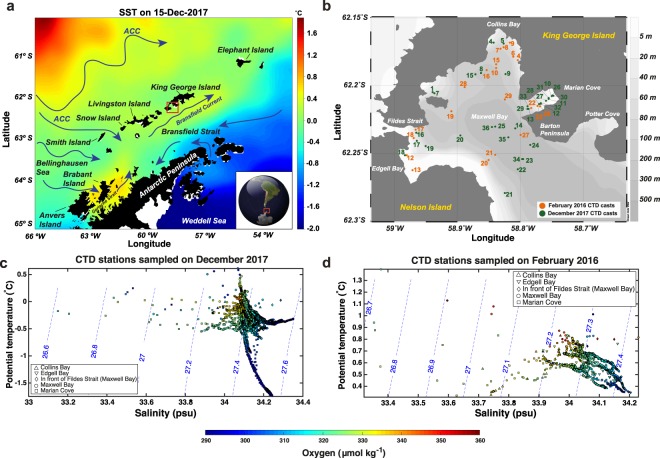
(**a**) Map of the Operational Sea Surface Temperature and Ice Analysis (OSTIA)^76^ at 1/20° resolution for the 15^th^ December 2017 including the geographical locations and main currents discussed in the paper. ACC: Antarctic Circumpolar Current. (**b**) Zoomed map (i.e. red square in 1a) of Maxwell Bay and tributary fjords with the hydrographic stations sampled during February 2016 (orange dots) and December 2017 (green dots). The software used to create the maps was Python 3.7.0 with Basemap and Ocean Data View 5.1.5^77^ (**c**) Theta-S diagram with contours of potential density anomaly (kg m^−3^) and the concentration of dissolved oxygen (µmol kg^−1^) colour coded from the CTD measurements accomplished in Maxwell Bay and tributary fjords in December 2017 and (**d**) February 2016.

King George Island (KGI) is found at the center of the South Shetland Islands (SSI) in West Antarctica (Fig. 1a). The SSI archipelago is separated from the West Antarctic Peninsula (WAP) by the Bransfield Strait and from South America by the waters of the Hoces Sea (i.e. Drake Passage). The broad and deep Maxwell Bay, also known as Fildes Bay, is one of the two main embayments in the western part of KGI, the other being Admiralty Bay. The mouth of Maxwell Bay is located at its southeastern sector and enables exchange (above a 430 m deep submarine sill) with the waters in the central basin of the Bransfield Strait. In contrast, the narrow and shallow Fildes Strait, located between Nelson Island and KGI, allows limited surface water exchange with the ocean to the north (Fig. 1b). Maxwell Bay is bordered by several smaller tributary bays and coves in which tidewater glaciers present active iceberg calving, specifically: Collins Bay, Marian Cove, Potter Cove and Edgell Bay.

High latitude bays and fjords are typically strongly stratified during the summer, with a layer of cold freshwater of riverine or glacial origin overlaying warmer salty water of oceanic origin^1,2^. The strong density gradient separating these layers tends to inhibit turbulent vertical mixing, decoupling the surface circulation (driven by the wind and buoyancy fluxes) from that below the halocline. The input of freshwater generates a pressure gradient that sets the surface layer in motion. This outward surface flow is compensated by an inward flow of oceanic waters at mid-depth (below the halocline). The resulting vertical velocity shear between the outgoing low salinity waters and the incoming saltier oceanic waters induces a net upward transfer of salinity and volume from the incoming oceanic waters via entrainment^3–5^. This circulation is known as estuarine circulation, also referred to as gravitational, baroclinic or buoyancy-driven circulation. The estuarine circulation can be masked, reinforced or even reversed by strong winds, tides and by remotely forced changes in the vertical density structure of the adjacent ocean^6–13^. When a submarine sill is present, deep, infrequently renewed, waters may form below sill level, while the intermediate waters found between sill level and the halocline may respond freely to external density variations^11^. As a result, the fact that the grounded ice front generally abuts intermediate waters means that changes in the vertical structure of the open ocean can propagate into the fjord and affect the glacial heat balance. Within the fjords that host tidewater glaciers, however, a number of processes can occur to modify the hydrographic structure through the generation of vertical motion. For example, ice-front melting and subglacial discharge often create rising cold fresh buoyant plumes, while fluvial discharge from side-entry glaciers or supraglacial meltwater contribute to create the fresh upper layer. The upwelling of subglacial meltwater plumes entrains saltier waters that partially mix with the plume, and the upwelling promotes the lateral advection of relatively warm oceanic waters^14–16^ toward the ice/ocean interface.

Several studies have examined the hydrographic structure and episodic intrusions of the relatively warm subsurface oceanic waters onto the WAP Shelf south of the SSI, including their transport across the shelf into coastal bays and fjords^17–23^. These studies took advantage of the proximity to the US Palmer (Anvers Island) and the UK Rothera (Adelaide Island) Stations. However, the intrusions of relatively warm oceanic waters into Maxwell Bay have not been previously examined, despite their possible great importance for delivering heat and nutrients to the Antarctic fjords found in this area.

In the SSI region, observational studies have described the hydrographic structure of the neighbouring Bransfield Strait^24–27^, its circulation^28,29^ and the variability of its deep waters^27–30^ on a larger spatial scale. Furthermore, a limited number of studies have described the local hydrography of individual fjords during the austral summer^31–33^. However, a description of the hydrographic structure and its variability at the spatial scale of Maxwell Bay is missing. This work aims to provide an updated and comprehensive picture of the hydrographic variability induced by tides, winds and the advance of the austral summer in the upper 200 m of the water column in Maxwell Bay (KGI). Furthermore, we have examined the fingerprint of relatively warm subsurface water intrusions into this delicate polar environment. The understanding and monitoring of these episodic intrusions into Antarctic fjords is of great importance for improving projections of future sea-level rise as well as for the evolution of primary productivity in Antarctic coastal waters.

## The Oceanographic Setting

The WAP region is heavily influenced by the southern reaches of the Antarctic Circumpolar Current (ACC)^34^ (Fig. 1a), whose waters are warmer and saltier than the Antarctic Shelf waters^19^. In other Antarctic regions, the ACC has limited access to the Antarctic Shelf due to the existence of subpolar gyres and/or a well-developed Antarctic Slope Current (ASC) that keep the ACC at distance from the shelf. There is no subpolar gyre in the region of study and a weak ASC flows westward only between the northern part of Elephant Island and the gap between Smith and Snow Islands^35,36^. This physical setting allows the ACC to reach the shelf break, providing a pathway for the intrusion of relatively warm subsurface waters onto the shelf^20,37^. The subsurface water mass transported within the ACC is Circumpolar Deep Water (CDW)^34^, commonly divided into Upper (UCDW) and Lower (LCDW) water masses. UCDW is more likely to intrude onto the Antarctic Shelf, although LCDW (colder and saltier than UCDW) has been found at the bottom of deep canyons cutting through the Antarctic Shelf in the southern part of the WAP^20,23,38,39^.

The UCDW carried by the ACC is characterized by salinity values ranging between 34.5 and 34.75 PSU, temperatures between 1.70 and 2.13 °C, low oxygen content (<195 μmol kg^−1^) and a high concentration of inorganic nutrients^19,35,40^. The UCDW has been found to be transported onto the Antarctic Shelf via upwelling favourable winds^41,42^, warm eddies detaching from the mean flow^20,23^, tidal mixing^43,44^ and coastally-trapped barotropic Kelvin waves^45^. Consequently, the UCDW represents a source of heat and nutrients into the WAP Shelf, where it progressively mixes with the colder and fresher shelf waters, sustaining the rich biological systems of the region^18,35^. As a result of this mixing, a colder (1.2–1.3 °C) and fresher (≥34.5 PSU) variety of UCDW is usually found over the continental shelf of the WAP between 150 m and 400 m^35^. This UCDW variety has been called WAP Shelf Water (WAP-SW)^39^ and, more frequently, modified UCDW (m-UCDW)^19,46^. The episodic intrusions of m-UCDW into the Antarctic Shelf have been suggested as the main driver of ice-shelf disintegration and tidewater glacier retreat around Antarctica^17,47–49^.

The surface waters found above the Antarctic Shelf during the austral summer are known as Antarctic Surface Waters (AASW)^35^. These waters are influenced by solar heating and meltwater inputs during the austral summer, presenting temperatures above 0 °C and salinities around 33.5 PSU^21^. The AASW layer presents a marked seasonal pattern, being colder and saltier from the austral fall to the austral spring^21,39^. During fall and winter, the AASW is cooled close to its freezing point (~−1.85 °C) while its salinity increases due to brine rejection during sea-ice formation^19^. This surface increase in density, together with increased wind-induced mixing, causes the seasonal pycnocline to disappear and the mixed layer to thicken. During spring and summer, remnants of the AASW cooled during the previous winter, known as Winter Water (WW), are usually found above the permanent pycnocline (~150 m) in the southern WAP, while WW occupies the entire mixed layer during the austral winter^19,50^. In the southern WAP, these WW remnants are characterized by temperatures ranging between −1.85 °C and −1.0 °C, salinities between 33.85 and 34.13 PSU and dissolved oxygen content between 282 and 326 μmol kg^−1^ ^35,51^. In contrast, in the central basin of Bransfield Strait there is no clear permanent pycnocline and WW convection reaches to the bottom^24,25^. As described more in detail below, we observed WW remnants also at the bottom of Marian Cove, a shallow-silled fjord (Fig. 1c,d). The AASW flowing into the Bransfield Strait from the Bellinghausen Sea and the Gerlache Strait is also known as Transitional Zonal Water with Bellinghausen influence (TBW)^28,52,53^.

The m-UCDW is thought to intrude into the Branfield Strait by 1) flowing northeast from the Gerlache Strait, through a trench between Anvers and Brabant Islands or 2) through the southwestern passages (located between Snow, Smith and Low Islands and the Antarctic Peninsula)^35,46,54^ and occasionally 3) flowing south between King George and Elephant Island^35,36,55^. Once inside the Bransfield Strait, m-UCDW is transported northeastward along the southern slope of the South Shetland Islands by the narrow (~10 km) Bransfield Current^28,55^. This is a gravity current caused by the Coriolis effect and the density difference (Bransfield Front) due to the intrusion of m-UCDW and AASW/TBW into the Bransfield Strait, which is dominated by the colder and relatively well ventilated Bransfield Strait Waters (BSW) (−1.62 °C, 34.58 PSU and 307.77 μmol kg^−1^). The BSW derive from the mixture of High (34.60 PSU) and Low (34.30 PSU) Salinity Shelf Waters at freezing point from the neighbouring Weddell Sea (HSSW/LSSW) with a small fraction of the relatively warmer (up to 0.56 °C) m-UCDW and Weddell Deep Water (WDW)^24–28,30,35^. BSW are also known as Transitional Zonal Water with Weddell Sea influence (TWW)^52,53^. Eastward of the Bransfield Front, a field of anticyclonic eddies caused by instabilities of the Bransfield Current^28^ enhances the mixing of m-UCDW and AASW/TBW with BSW/TWW^56^.

The internal Rossby radius of deformation typical of these latitudes (~10 km) is too large for the Coriolis effect to play a significant role in the circulation of the tributary fjords of Maxwell Bay. However, it has been shown to influence the local circulation inside Maxwell Bay^31^ where the width of Maxwell Bay is larger than the internal Rossby radius of deformation^35,56^.

## Results and Discussion

For Antarctic coastal waters, variability over daily and weekly timescales (induced by winds, tides and mesoscale activity) can be as large as that of the seasonal cycle^38,39^. Furthermore, the temperature of the seasonal cycle increases with distance north from the Antarctic coast^39^. In this section, we first discuss the effects of tides and winds on the hydrography of Maxwell Bay and tributary fjords. Secondly, we analyse the oceanographic changes caused by the advance of the austral summer (intra-seasonal cycle). Finally, we describe the hydrographic changes induced by warm subsurface water intrusions into Maxwell Bay.

### Variability induced by tides and winds

Satellite measurements indicate that the surface wind speed during the austral summer in the SSI region ranges from 4 to 8 m/s^57^. In neighbouring Admiralty Bay, the surface water circulation appears to be dominated by wind whenever it exceeds approximately 4 m/s^32,58^ and otherwise is driven by the tides^58^. However, this threshold is just an approximation given the changing nature of tides during the spring/neap tidal cycle. Due to its geographical proximity, a similar response to wind and tide forcing may be expected to occur in Maxwell Bay and its tributary fjords. Besides driving surface circulation, winds can also affect deeper in the water column as wind-driven upwelling events break the stratification induced by meltwater discharge^31,32^.

Tides in the central and western basins of the Bransfield Strait were found to be stronger than the geostrophic flow (below the surface Ekman layer) with velocities up to 40 cm/s^59^. Tides are involved in water mass transports (and mixing) between Bransfield Strait and Maxwell Bay and viceversa, as as well as between Maxwell Bay and its tributary fjords^31,33,58^. In the following subsections, we examine the influence of the tides and winds in the variability of the oceanographic properties measured at Maxwell Bay and the Marian Cove and Collins Bay tributaries.

#### Maxwell bay

Maxwell Bay is a deep fjord (~500 m) located in the western part of KGI and delimited on its western part by Nelson Island. Its longitudinal axis (~14 km) is oriented in the NW-SE direction^60^. From measurements taken at Bellinghausen station, the mixed semi-diurnal tide has a mean tidal amplitude of 103 cm, with a syzygial (spring) amplitude of 146 cm and a quadrature (neap) amplitude of 42 cm^58^.

**Figure 2:**
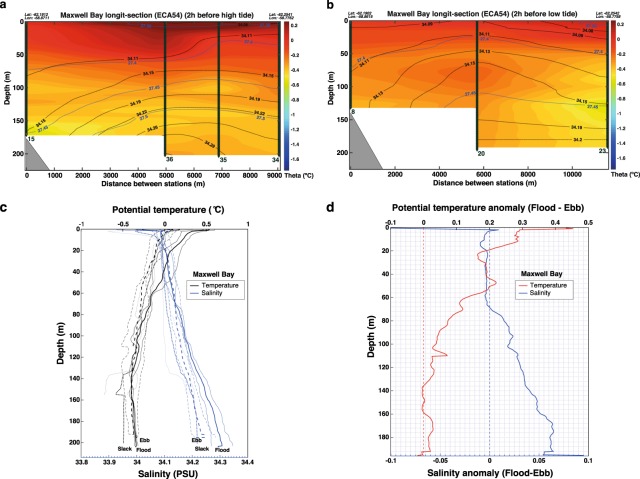
Longitudinal section of Maxwell Bay obtained in December 2017 showing potential temperature (°C, colored), salinity (PSU, black contours) and potential density anomaly (kg m^−3^, blue contours) during (**a**) flood tide and (**b**) ebb tide. The position (triangles) and number corresponding to each CTD cast are also shown. (**c**) Mean profiles (thick lines) and standard deviation (thin lines) of potential temperature (°C, black lines) and salinity (PSU, blue lines) from the CTD casts sampled in Maxwell Bay in December 2017 at flood tide (solid lines), slack tide (dotted lines) and ebb tide (dashed lines). (**d**) Anomaly of the average profiles (flood tide minus ebb tide) of potential temperature (°C, red line) and salinity (PSU, blue line).

The hydrography observed along the longitudinal axis of Maxwell Bay in December 2017 varied over both the tidal phase and location (Fig. 2). Toward the head of Maxwell Bay, the isopycnal 27.4 deepened by 20 m from ebb tide to flood tide. In addition, a 70 m thick surface layer with low salinities (<34.11 PSU) is observed during flood tide toward the head of Maxwell Bay, consistent with an increased retention of meltwater in the upper layers during flood tide (Fig. 2a). In contrast, toward the mouth of Maxwell Bay, both the 27.4 isopycnal and the 34.11 isohaline shoal during flood tide, indicating that a thinner surface meltwater plume leaves Maxwell Bay during flood tide (between casts 34 and 36) compared to ebb tide (between casts 20 and 23, Fig. 2b). A thin and fast flowing freshwater surface plume has also been observed in Alaskan fjords during flood tide under calm winds^16,61^.

Meltwater plumes are enhanced by sustained above freezing conditions^31^. The local air temperature was below freezing, however, for the 33 h prior to the casts sampled at flood tide, only exceeding freezing for the 3 h prior to the casts sampled at ebb tide. Consequently, it is concluded that air temperatures cannot explain the observed accumulation of meltwater in the head of Maxwell Bay during flood tide. Similarly, the weakness of the winds (<4 m/s) during the sampling period (and the previous 6 h), suggests that they played no significant role on the hydrographic structure shown in Fig. 2a,b. The weak winds also make unlikely that a seiche caused the observed changes.

All the casts shown in Fig. 2b were sampled within the last 2 h before low tide. Similarly, in Fig. 2a the casts 34 to 36 were taken within the last 2 h before high tide but the cast 15 was taken 5 h before high tide. Due to logistic limitations, cast 8 (Fig. 2b) and cast 15 (Fig. 2a) were sampled on a different day than the rest of the casts shown in their respective transects. Consequently, the portion of these transects between casts 15 and 36 (Fig. 2a) and between casts 8 and 20 (Fig. 2b) should be regarded with caution. Nevertheless, the deepening of the isohaline 34.11 (and isopycnal 27.4) at flood tide described between casts 36 and 15 (sampled 11 days apart) is also observed between casts 35 and 36 (sampled 30 minutes apart), suggesting that the meltwater retention observed towards the head of Maxwell Bay during flood tide might be a recurring feature under light winds and similar surface air temperature conditions.

In the Methods Section, we discuss how a barotropic tidal current of approximately 40 cm/s can explain this feature. Barotropic tidal currents in the Bransfield Strait have mean amplitudes of 30–40 cm/s^59^. Nevertheless, tidal currents may strengthen when funneled through a narrow passage, reaching for instance up to 76 cm/s when passing through the Neptuno Bells in the neighbouring Deception Island^62^. Similarly, a strengthening of the tidal currents is to be expected inside Maxwell Bay when passing through its narrowest part (between the Barton Peninsula and Nelson Island, Fig. 1b), which communicates the mouth of Maxwell Bay with its head (where the meltwater retention has been observed at flood tide).

In the following, we discuss the plausibility of other mechanisms that could have caused the observed meltwater accumulation toward the head of Maxwell Bay (under light wind conditions). These mechanisms are: 1) fluctuations of the Bransfield Current; 2) coastally trapped waves (CTW); 3) internal tide; and 4) tidal straining. The first two mechanisms involve the geostrophic transport of meltwater from the neighbouring Nelson Island glaciers toward the head of Maxwell Bay. Nonetheless, CTW were found to be present only below 60 m inside the inner bay of Deception Island^63^ and, assuming a similar behaviour of CTW in Maxwell Bay, we do not expect CTW to play a role in the observed meltwater accumulation in the upper 70 m of the water column.

The possibility that the observed water column disturbance could result from internal waves driven by the barotropic tide passing over the ~500 m sea bottom difference between the mouth of Maxwell Bay and the Bransfield Strait, cannot be completely discarded. Internal tides often have amplitudes at depth around 20 m that influence the vertical distribution and mixing of oceanographic properties, and have recently been observed to drive large salinity and temperature fluctuations in the inner bay of Deception Island^63^. Consequently, the internal tide could have induced the observed deepening of the isopycnal 27.4 and isohaline 34.11.

Finally, tidal straining is caused by the vertical gradient of the tidal current (stronger surface flow) in the presence of an along-fjord density gradient^64^. During the ebb, this process induces an increased stratification of the upper part of the water column, as fresher surface waters are advected faster down-fjord over more saline water, resulting in a thin and fast flowing low salinity layer. The reverse situation occurs during flood tide, with the vertically sheared flood current moving faster up-fjord in the surface layer, resulting in a destratification of the upper layer close to head of the fjord, reflected by the formation of a thick layer of low salinity waters^64,65^. Given that we observe an along-fjord density gradient (Fig. 2a,b), tidal straining could contribute to the meltwater retention feature described in Fig. 2a as long as the barotropic tide current is vertically sheared. However, even though tidal straining has been described (and modelled) for several estuaries^9,10,64,66–69^, it has yet to be studied in fjords.

Figure 2c shows the mean profiles of salinity and potential temperature from the casts sampled in the central and deepest part of Maxwell Bay in each tidal phase (flood tide: casts 34 to 36; ebb tide: casts 20 to 24 and slack tide: casts 14 and 25). These average profiles provide an idea of the mean change in hydrographic properties at different tidal phases in the central and deepest part of Maxwell Bay. At slack tide (end of ebbing) the upper 70 m were on average fresher (0.021 PSU) and colder (0.16 °C) than during flood tide. However, this freshening was possibly enhanced by the strong winds blowing during the sampling of cast 25 (~10 m/s, southwesterly wind) and cast 14 (~4.5 m/s, northerly wind), by moving surface meltwater toward these casts.

Figure 2d shows the change in potential temperature and salinity between the average profile at flood tide and the average profile at ebb tide (from Fig. 2c) in the central and deepest part of Maxwell Bay. According to Fig. 2d, the upper 70 m are on average 0.20 °C warmer and 0.005 PSU fresher during flood tide than during ebb tide (Fig. 2c,d). Note that this depth range is only slightly fresher during flood tide because we are comparing the mean profiles at different tidal phases from the central and deepest part of Maxwell Bay and the meltwater retention is larger the closer to the head of Maxwell Bay (Fig. 2a). The largest difference in water properties between flood and ebb tide was found in the upper 2 m, being warmer and fresher (+0.45 °C/−0.1 PSU) during the flood tide sampling, consistent with a thinner and fresher surface meltwater plume leaving Maxwell Bay during the flood tide. Its warmer temperature may be explained by the absorption of solar radiation in this surface layer.

Below 70 m, average casts are saltier (up to 0.07 PSU) and warmer (up to 0.1 °C) during flood tide compared to ebb tide, although progressively colder with depth (Fig. 2d). This suggests that the flood tide enhances the intrusion into Maxwell Bay of a mixture of salty and warm m-UCDW with the colder and less salty BSW. This is supported by previous studies that documented the role of the residual tidal flow in driving water exchange in the inner bay of Deception Island^62,70^. In the absence of winds, remote forcing or a strong estuarine circulation, the residual flow in deep fjords (>100 m) is tidally driven and three-layered, with outflowing surface and bottom layers and an inflowing intermediate layer^13,71^. While our dataset does not include current velocity data, the profile anomalies shown in Fig. 2d represent a proxy for the direction of the tidal residual flow, with negative anomalies in salinity indicating fresher up-fjord waters and hence a down-fjord residual velocity, and similarly positive salinity anomalies indicating an up-fjord residual current. In this sense, the profile of salinity anomalies in Fig. 2d suggests a two-layered residual flow with down-fjord velocity in the upper 60–70 m of the water column and up-fjord residual flow from 70 to 190 m at least. However, given that we could not sample the water column deeper than 200 m due to logistic limitations, a three-layered residual flow in Maxwell Bay cannot be ruled out, with down-fjord residual flow at the bottom layer during the period of our measurements.

While tides have been shown to play an important role in the cross-shelf intrusions of m-UCDW around Antarctica^43,72^; there are other plausible mechanisms that can induce (or strengthen) m-UCDW intrusions in Maxwell Bay like: (1) CTW produced by wind bursts; (2) fluctuations of the density field (baroclinic pumping) in the Bransfield Strait outside Maxwell Bay; (3) eddies detaching from the Bransfield Current; and (4) wind induced upwelling of m-UCDW at the mouth of Maxwell Bay (albeit unlikely given the weak winds during the field work). Nevertheless, a detailed investigation of the forcings behind these intrusions is beyond the scope of this study.

**Figure 3:**
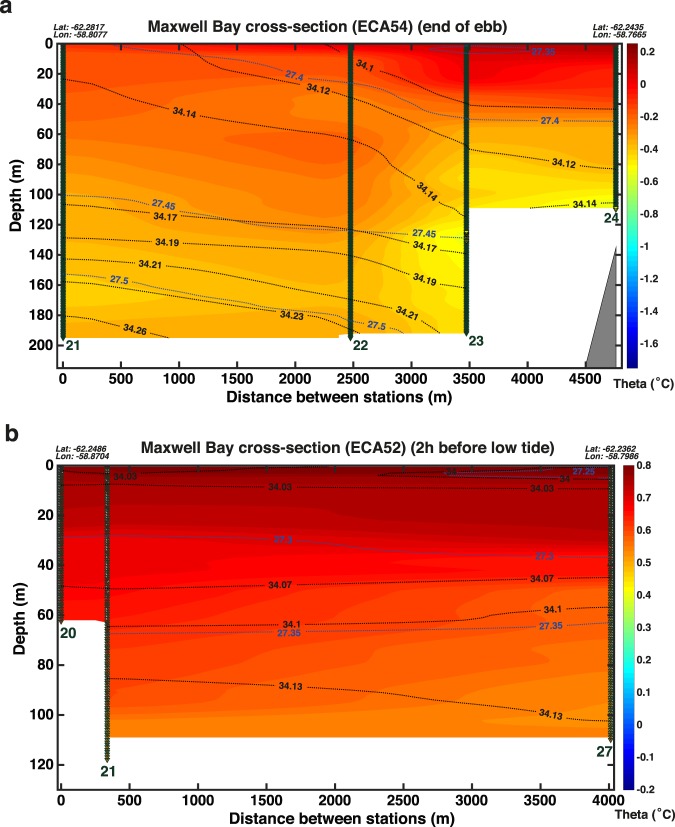
Cross section of Maxwell Bay showing potential temperature (°C, coloured), salinity (PSU, black contours) and potential density anomaly (kg m^−3^, blue contours) during ebb tide obtained in (**a**) December 2017 and (**b**) February 2016. The position (triangles) and number corresponding to each CTD cast are also shown.

A transect across Maxwell Bay from Nelson Island to Barton Peninsula in KGI revealed isopycnals and isohalines in the upper 190 m shoaling toward the SW in December 2017 (Fig. 3a). Furthermore, between casts 23 and 24 (NE) the upper 50 m have salinity values below 34.10 PSU, which contrasts with the saltier waters (up to 34.15 PSU) found between casts 21 and 22 (SW) (Fig. 3a). All the casts shown in Fig. 3a were accomplished within 1 h 15 mins during the ebb tide. Supposing that this tilt of the isopycnals was a permanent feature, this would imply a geostrophic flow leaving Maxwell Bay for at least the upper 100 m (between casts 23 and 24) and the upper 190 m (between casts 21 and 23). Winds during the sampling of this transect (and the previous 5 h) were too weak (~3 m/s, southwesterlies) to have caused the tilt of isopycnals and isohalines described above.

A similar cross-section was accomplished under light wind conditions in February 2016 (Fig. 3b) with clearly warmer and fresher waters in the upper 100 m. This section shows a much weaker tilt (shoaling toward the SW) of isopycnals (upper 40 m) and isohalines (upper 5 m). For Fig. 3b, casts 20 and 21 where sampled 30 mins apart during the ebb tide while cast 27 was taken 4 h later than cast 21 with the flood tide. Chang and collaborators^73^ suggested that a cyclonic circulation might be caused by the Coriolis effect in the sectors of Maxwell Bay wider than the first baroclinic radius of deformation. This would imply a fraction of the water transported by the northeastward flow of the Bransfield Current entering Maxwell Bay through the southwestern part of its mouth and leaving it with a large influence of meltwater through the northeastern part of its mouth.

#### Marian cove

Marian Cove is a narrow tributary fjord of Maxwell Bay with its ~4.5 km longitudinal axis running in the NE-SW direction. Marian Cove is delimited to the north by the Weaver Peninsula, to the south by the Barton Peninsula and to the northeast by a tidewater glacier (Fig. 1b). The ~1.5 km wide mouth of Marian Cove is located in its southwestern sector, opening to Maxwell Bay over a 50 m deep sill. The presence of this sill conditions the characteristics of the tide within Marian Cove^31,60^. Along its longitudinal axis, Marian Cove is divided into three relatively deep basins separated by 70 m deep sills, with a maximum basin depth of about 135 m in the basin located in the proximity of the ice-cliff at the head of the fjord^31^. In this fjord, the mixed semi-diurnal tide has a mean tidal amplitude of 1.5 m, with a syzygial tide amplitude of 2.8 m^60^.

**Figure 4:**
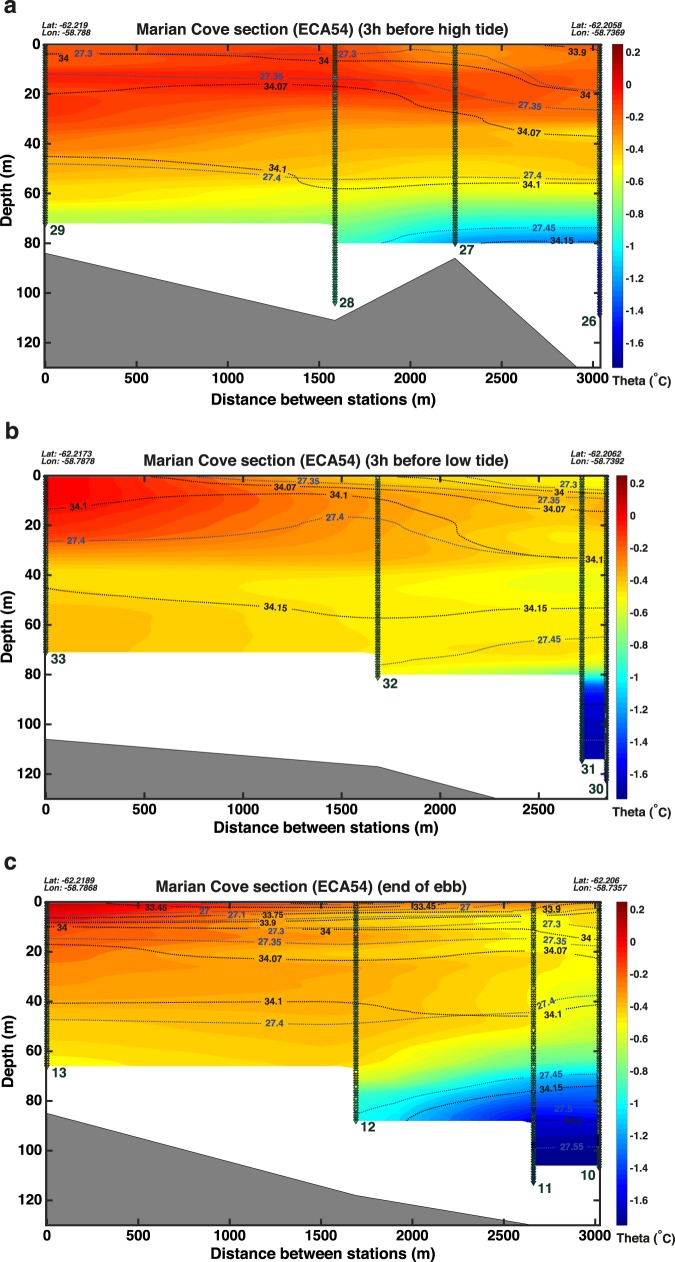
Longitudinal section of Marian Cove obtained in December 2017 showing potential temperature (°C, coloured), salinity (PSU, black contours) and potential density anomaly (kg m^−3^, blue contours) during (**a**) flood tide, (**b**) ebb tide and (**c**) slack tide. The position (triangles) and number corresponding to each CTD cast are also shown.

**Figure 5:**
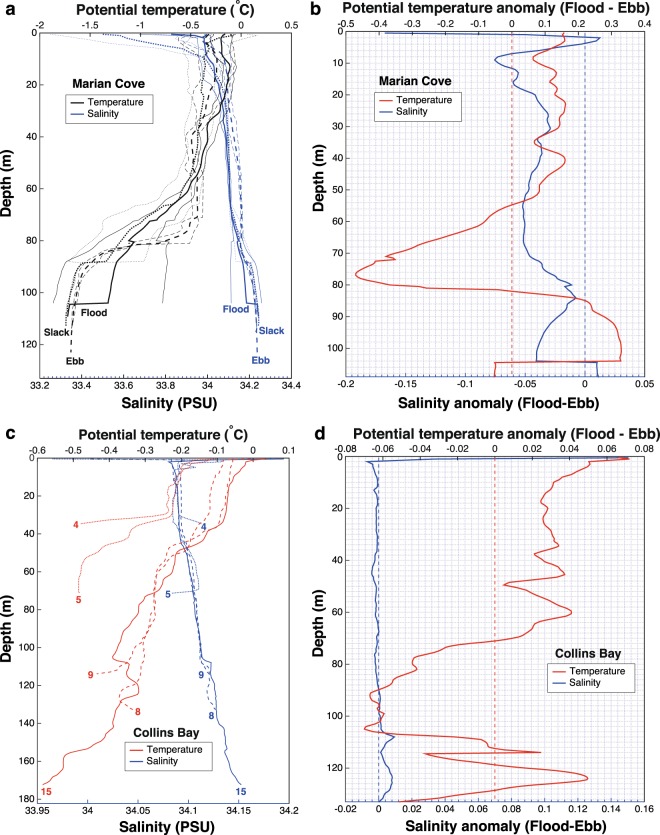
(**a**) Mean profiles (thick lines) and standard deviation (thin lines) of potential temperature (°C, black lines) and salinity (PSU, blue lines) from the CTD casts sampled in Marian Cove in December 2017 at flood tide (solid lines), slack tide (dotted lines) and ebb tide (dashed lines). (**b**) Anomaly of the average profiles (flood tide minus ebb tide) of potential temperature (°C, red line) and salinity (PSU, blue line) sampled in Marian Cove. (**c**) Profiles of potential temperature (°C, red lines) and salinity (PSU, blue lines) from the CTD casts sampled in Collins Bay in December 2017 at flood tide (solid lines), slack tide (dotted lines) and ebb tide (dashed lines). (**d**) Anomaly of the average profiles (flood tide minus ebb tide) of potential temperature (°C, red line) and salinity (PSU, blue line) sampled in Collins Bay.

Vertical sections were sampled along the longitudinal axis of Marian Cove at different tidal phases in December 2017 (Fig. 4). The casts shown in each transect (Fig. 4) were all accomplished within 1 h 20 min during the corresponding tidal phase. As is commonly the case for small fjords like Marian Cove, water properties vary with distance to the fjord’s head: for the same tidal phase, the casts located closer to the head (casts 10 and 27) are relatively colder and fresher than the casts sampled farther away (casts 13 and 29). During the flood tide, the isopycnals (27.30, 27.35) and isohalines (34.01, 34.07) were found to deepen toward the head (cast 26) of Marian Cove, due to a surface layer with salinities below 34.07 of ~40 m thick close to the head of the fjord (between casts 27 and 26) (Fig. 4a). The retention of meltwater in the surface layer at the head of Marian Cove was probably induced by the flood tide, helped by a persistent westerly-southwesterly wind (~9 m/s) blowing during the previous 5 h. The downward tilt of isopycnal 27.3 and isohaline 34 between casts 26 and 27 is consistent with wind-driven downwelling in the head of Marian Cove. The internal tide and tidal straining may have also played a role in the formation of this low salinity surface layer. During the ebb tide, the isopycnals and isohalines were shallower in the entire vertical section (Fig. 4b). For example, the isopycnals 27.4 and 27.35 and isohalines 34.1 and 34 are found 20–25 m shallower compared to flood tide in the sector close to the head of Marian Cove. This indicates that meltwater leaves Marian Cove aided by the ebb tide, with its freshwater signature clearly observed in the upper 20 m of the water column. The westerly-southwesterly wind (~9 m/s), blowing during and in the previous 18 h to the casts sampled at ebb tide, is expected to partially compensate the ebb flow and can be detected in the deepening of isopycnal 27.35 and isohaline 34.07 toward the head of Marian Cove (between casts 32 and 30) (Fig. 4b). During the slack tide after the ebb (casts 10 to 13), the upper 10 meters were colder and fresher than during the flood or the ebb (Figs. 4 and 5a). This can be explained by the northwesterly wind that blew at speeds ranging from 4 to 10 m/s during the previous 17 h, reinforcing the ebb and the estuarine circulation, but also inducing a weak upwelling as indicated by the shoaling by ~5 m of the shallower isopycnals (<27.20) and isohalines (<33.90) close to the ice front (between cast 11 and 10, Fig. 4c). The water column between 20 and 40 m depth was on average 0.16 °C colder and 0.009 PSU saltier during slack than during flood.

Comparing the average profiles between flood tide and ebb tide, we found that the upper 55 m are on average 0.12 °C warmer and 0.043 PSU fresher during flood tide (casts 27 to 29) than during ebb tide (casts 30 to 33) (Fig. 5a,b). Between 55 and 82 m depth, there is a layer of water that was up to 0.47 °C colder and 0.052 PSU fresher at 76 m during flood tide than during ebb tide (Fig. 5b), probably related with the shoaling and mixing of Winter Waters from below, enhanced by internal waves (this will be further discussed at the end of this subsection). In contrast, the layer between 80 and 103 m depth was 0.23 °C warmer and 0.026 PSU fresher during the flood than during the ebb (Fig. 5b).

As discussed for Maxwell Bay, the profile anomalies shown in Fig. 5b could be used as a rough proxy of the direction of the tidal residual flow. The profile of salinity anomalies in Fig. 5b suggests a two-layered residual flow, with down-fjord flow in most of the water column and up-fjord flow below 104 m depth. This pattern of flow indicates a strong estuarine circulation driven by large meltwater discharges from the tidewater glacier found at the head of Marian Cove. However, a weak three-layered pattern can be distinguished superimposed on the strong estuarine circulation, depending on the magnitude of the salinity anomalies and on the sign of the anomalies observed in potential temperature (Fig. 5b). In this sense, it seems that there is a weak up-fjord residual flow that compensates part of the estuarine flow in the upper part of the water column (between 12 and 54 m depth) and in the bottom part of the water column (below 80 m depth) and a down-fjord residual flow in an intermediate layer (between 54 and 80 m depth). Such three-layered flow in strongly stratified estuaries has been observed to occur via tidal straining^10,69^.

Air temperatures above freezing were measured during the previous hours to the casts sampled at ebb (previous 9 h), flood (previous 12 h) and slack (previous 18 h) tide in Marian Cove. Consequently, the observed changes in the freshwater plume distribution in our dataset can be explained by the tide phase and, to a lesser extent, by the wind forcing. However, the internal tide, tidal straining and fluctuations of the density field outside Marian Cove may have played a role in the hydrographic structure observed.

In the following we describe how remnants of the cold Winter Water (WW) mix with warmer and saltier waters at the bottom of the inner basin in Marian Cove. At the beginning of the austral summer, the inner basin is mainly composed of WW, as it presents a very cold temperature (between −0.5 and −1.7 °C), with salinity (~34.20 PSU) and oxygen (~290 μmol kg^−1^) values corresponding to slightly modified WW (Fig. 1c). Our results indicate that this WW migrates in depth and mixes during the tidal cycle. During the ebb, the WW moves upwards by ~20 m and toward the mouth of Marian Cove (Fig. 4b,c), and mixes with the overlying warmer and fresher waters, as indicated by the colder waters found between 40 and 80 m at the end of ebbing between casts 12 and 10 (Fig. 4c). This upward mixing of WW can still be appreciated during flood tide, as a cold-water tongue (−0.6 to −0.9 °C) spreading along the entire fjord axis between 60 and 75 m depth (Fig. 4a). This feature is probably explained by the action of the internal tide generated when the barotropic tide current encounters the submarine sills present in Marian Cove. Internal tides can modify the distribution of oceanographic properties in the water column and their breaking induces mixing. In fact, the presence of WW in Marian Cove by the end of the summer is greatly reduced (Fig. 1d, see the next section). Consequently we propose that, over the course of the austral summer, successive tidal cycles progressively erode the remnants of WW found in the bottom of the inner basin in Marian Cove by enhancing its mixing with relatively warmer waters. This mixing may also be enhanced by the up-fjord flow induced at the bottom layer during the flood tide (Fig. 5b).

#### Collins Bay

Collins Bay is a relatively broad (~3 km wide at its mouth) fjord located in the northern sector of Maxwell Bay, with its coastline completely occupied by tidewater glaciers. Its longitudinal axis runs along a ~3 km line in the N-S direction. Its depth ranges from 100 m at its mouth (southern part) to 145 m in the basin to the northeast. Given its proximity to Bellinghausen station (less than 12 km), a mixed semi-diurnal tide is also expected for in Collins Bay.

Here again the distance to the fjord’s head explains much of the horizontal distribution of water properties, with the casts closer to the head (4 and 5) colder than those at the mouth of the bay (15, 8 and 9) (Fig. 5c). Casts 8 and 9 were taken 20 minutes apart while cast 15 was taken 3 h later than cast 9. These casts were taken 2 days after the sampling of casts 4 and 5 due to changing weather conditions. Casts 4 and 5 were sampled (20 minutes apart) during slack tide at the head of Collins Bay.

Cast 5 was slightly warmer and fresher than cast 4 located to its west (Fig. 5c). The observed density change is consistent with a wind-induced downwelling on the northeastern side of the Bay, in agreement with the strong (~11.5 m/s) westerly-southwesterly wind blowing during the sampling of Collins Bay on that day. However, other plausible causes of the observed changes between casts 4 and 5 are differential glacial melting, surface exchange or changes in the alongshore flow.

The upper 70 m of the water column in Collins Bay are on average 0.03 °C warmer during flood tide (−0.13 °C) compared to ebb tide (−0.16 °C), being the coldest (−0.33 °C) at slack tide (end of ebb) (Fig. 5c). Salinity did not change significantly between casts (Fig. 5d). During both flood and ebb tide, there was a light northerly wind (~4 m/s), which blew, stronger the earlier in the previous 16 h (up to 10 m/s). The northerly wind promotes the spreading of the surface freshwater plume away from the head of Collins Bay (i.e. away from the glacier cliffs). However, this wind was lighter in the 5 h previous to our measurements, and the increased meltwater content observed during flood tide inside Collins Bay suggests that this wind did not drive the surface low salinity water outside of Collins Bay.

The direction of the tidal residual flow can be inferred from the profile anomalies shown in Fig. 5d. Here, the profile of salinity anomalies suggests a two-layered residual flow, with a down-fjord flow in most of the water column and up-fjord flow below 100 m depth. This pattern of flow indicates a dominant estuarine circulation, which is to be expected in the austral summer given the glaciated coastline of Collins Bay. Similarly to Marian Cove, a three-layered pattern can be inferred (superimposed on the estuarine circulation) when considering the sign of the anomalies observed in potential temperature (Fig. 5d). The anomalies in potential temperature suggest an up-fjord residual flow (compensating part of the estuarine flow) in the upper 70 m and in the bottom layer (below 106 m depth) and a down-fjord residual flow in an intermediate layer (between 70 and 106 m depth). Given that Collins Bay is strongly stratified during the austral summer, the presence of tidal straining could explain this flow pattern^10,69^.

Air surface temperatures were above freezing during the previous 13 h to the casts obtained during flood and ebb tide, being higher in the 3 h prior to sampling at ebb tide (0.7–0.9 °C) than during flood tide (0.3 °C). Therefore, it may be concluded that air surface temperature (through its impact on glacier melting) was not responsible for the upper 70 m being slightly fresher during flood tide (Fig. 5d). Due to the effects of a persistent northerly wind and to the timing of the sampling relative to the tidal phase, the increased retention of meltwater discharges expected during flood tide inside Collins Bay is not as clearly observed as in Maxwell Bay. However, the observed hydrographic setting could also be influenced by the internal tide, by tidal straining and by fluctuations in the density field outside Collins Bay.

### Intraseasonal cycle

**Figure 6:**
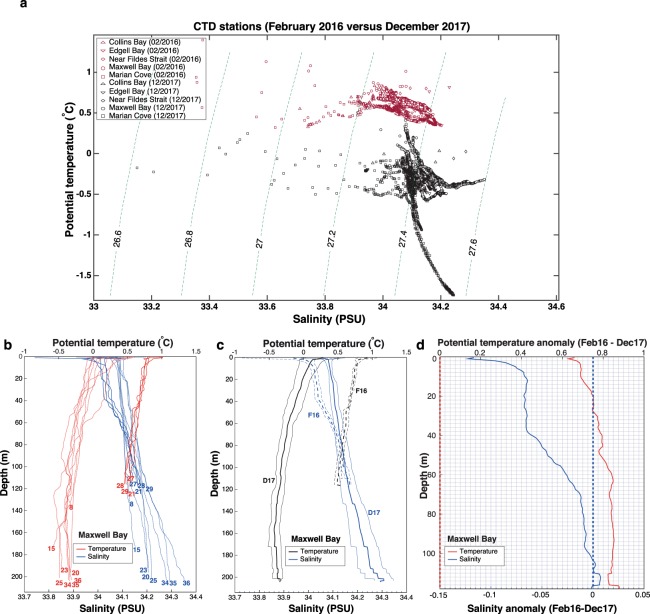
(**a**) Theta-S diagram with contours of potential density anomaly (kg m^−3^) from the CTD measurements accomplished in Maxwell Bay and tributary fjords in December 2017 (black symbols) and February 2016 (maroon symbols). (**b**) Profiles of potential temperature (°C, red lines) and salinity (PSU, blue lines) from all the CTD casts sampled in Maxwell Bay in December 2017 (dotted lines) and in February 2016 (solid lines). (**c**) Mean profiles (thick lines) and standard deviation (thin lines) of potential temperature (°C, black lines) and salinity (PSU, blue lines) from all the CTD casts sampled in Maxwell Bay in December 2017 (solid lines) and February 2016 (dashed lines). (**d**) Anomaly of the average profiles (February 2016 minus December 2017) of potential temperature (°C, red line) and salinity (PSU, blue line) sampled in Maxwell Bay.

In King George Island, meltwater discharges increase during the austral summer, following the average surface air temperature, which is 1.1 °C in December and peaks with 2.2 °C in February^60^. The buoyancy gain through warming and freshening of the surface layer enhances the stratification of the water column, leading to the formation of a shallow seasonal pycnocline^35^. Wind-induced upwelling events can temporarily break this seasonal pycnocline^31^. The surface ocean warms in contact with a warmer low-atmosphere and by the larger doses of solar radiation received during the summer. In Maxwell Bay, the integrated monthly solar radiation is larger than 400 MJ/m^2^ from November to January; while it decreases below 100 MJ/m^2^ per month between May and August^74^. The freshening is due to both the melting of sea-ice and to meltwater discharges from numerous tidewater glaciers and the rivulets that form during the summer. Furthermore, as the austral summer progresses, waves, winds and tides, progressively mix the surface buoyant layer down the water column, eroding from above the remnants of WW, characterized by a temperature minimum found around 100 m depth^35^. WW remnants are also eroded from below year-round by the mixing with m-UCDW (mixed itself with BSW) as indicated by the relatively low oxygen values found in the WW (Fig. 1c,d) with regard to their saturation value (~360 μmol kg^−1^). The fact that the two field campaigns took place at the beginning (early December 2017) and end (mid February 2016) of summer, provides the opportunity to examine the hydrographic changes that take place along this season. These results are likely representative, but it must be acknowledged that interannual variability could play a role in the variability observed between both campaigns. From a preliminary analysis of the Theta-S diagram in Fig. 6a, it is clear that the water column is warmer and fresher at the end of the austral summer (February) than at the beginning (December) in Maxwell Bay and its tributary fjords, with the exception of Marian Cove, in which the salinity does not change appreciably above isopycnal 27.2 (Fig. 6a).

#### Maxwell Bay

The vertical profiles obtained in Maxwell Bay from casts both at flood and ebb tide under different wind conditions in February 2016 and December 2017 are shown in Fig. 6b. It is clear that the variability induced in the water properties by the tidal phase, the oscillation of the density field and the blowing winds is of smaller magnitude than the intraseasonal hydrographic changes due to the progression of the summer (i.e. the more advanced is the summer the warmer and fresher is the upper-part of the water column). February casts (solid lines) are warmer and fresher than December casts (Fig. 6b,c), in good agreement with the changes inferred from Fig. 6a. Specifically, the upper 105 m are on average 0.79 °C warmer and 0.039 fresher in February than in December (Fig. 6d). The intraseasonal freshening is more intense in the upper 20 m, being on average 0.074 PSU fresher, while warming is more intense at depth, being on average 0.84 °C warmer between 60 and 118 m depth (the maximum depth of February casts) (Fig. 6d). The relatively warm (0.5 °C) and salty waters (up to 0.007 PSU saltier) found between 105 and 116 m depth in February suggest a larger presence of m-UCDW, which is not related with the seasonal forcing in the southern WAP^21,39^ although it might be in Maxwell Bay (and Bransfield Strait) due to the seasonality of the Bransfield Current, which transports this water mass in the subsurface^28^.

#### Collins Bay

**Figure 7:**
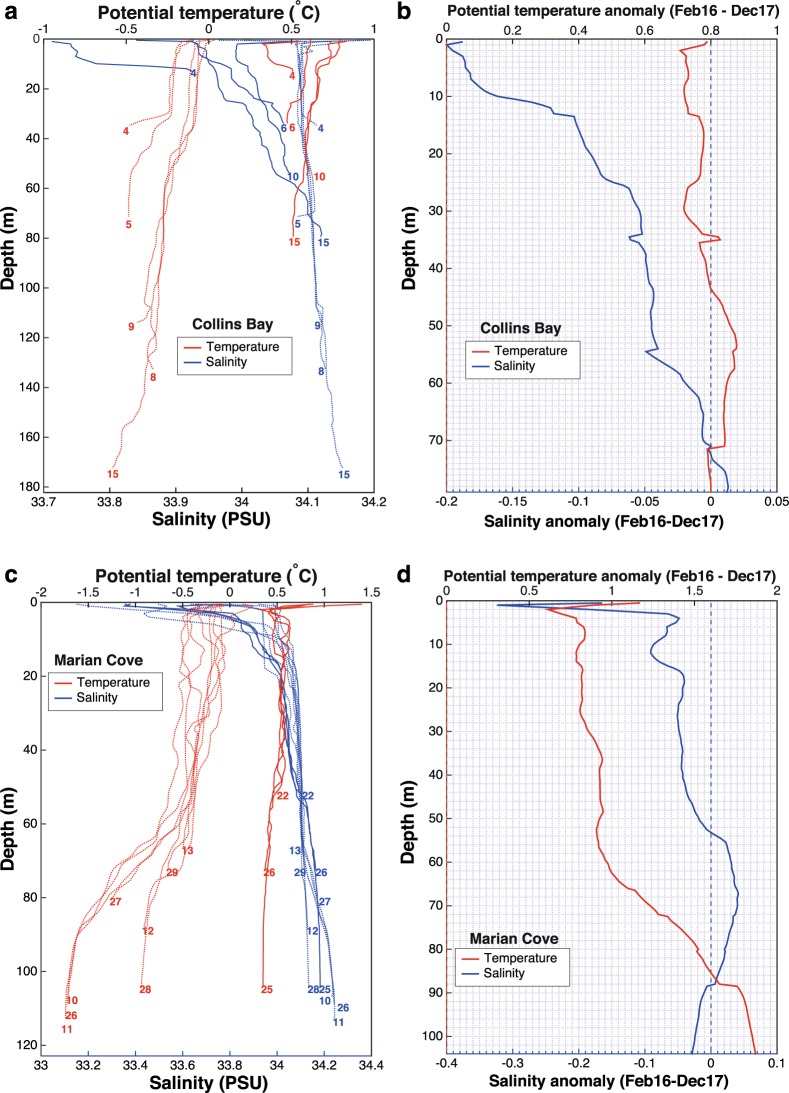
(**a**) Profiles of potential temperature (°C, red lines) and salinity (PSU, blue lines) from all the CTD casts sampled in Collins Bay on December 2017 (dotted lines) and on February 2016 (solid lines). (**b**) Anomaly of the average profiles (February 2016 minus December 2017) of potential temperature (°C, red line) and salinity (PSU, blue line) sampled in Collins Bay. (**c**) Profiles of potential temperature (°C, red lines) and salinity (PSU, blue lines) from all the CTD casts sampled in Marian Cove in December 2017 (dotted lines) and February 2016 (solid lines). (**d**) Anomaly of the average profiles (February 2016 minus December 2017) of potential temperature (°C, red line) and salinity (PSU, blue line) sampled in Marian Cove.

Similarly to Maxwell Bay, its tributary Collins Bay shows clearly a warmer and fresher water column in February than in December (Fig. 7a). Specifically, the upper 80 m are on average 0.79 °C warmer and 0.063 PSU fresher (up to 0.2 PSU in the upper 2 m) (Fig. 7b). Saltier waters (up to 0.013 PSU) were found close to the bottom of Collins Bay in February, suggesting a larger influence of m-UCDW below 72 m, as found for Maxwell Bay (Fig. 7b).

#### Marian Cove

As for Collins Bay, the casts obtained in Marian Cove in February were warmer and fresher than the casts obtained in December (Fig. 7c). In this fjord the entire water column was on average 1.14 °C warmer and 0.022 PSU fresher in February; being the upper 54 m up to 0.323 PSU fresher. However, between depths of 54 and 88 m, the water column is up to 0.041 PSU saltier (and 1.65 °C warmer) in February than in December, being again fresher (up to 0.028 PSU) below 88 m in February (Fig. 7d). This feature might be caused by a larger presence of m-UCDW in Marian Cove during February casts, which were only sampled during the flood tide (while December casts were sampled at flood, ebb and slack tide).

### Modified Upper Circumpolar Deep Water intrusions in Maxwell Bay

Most of the m-UCDW entering the Bransfield Strait does so through its southwestern sector^35^. From there, m-UCDW is transported northeast by the narrow Bransfield Current, flowing along the southern slope of the South Shetland Islands where it may be further modified by mixing with the much colder BSW^28,55^.

**Figure 8:**
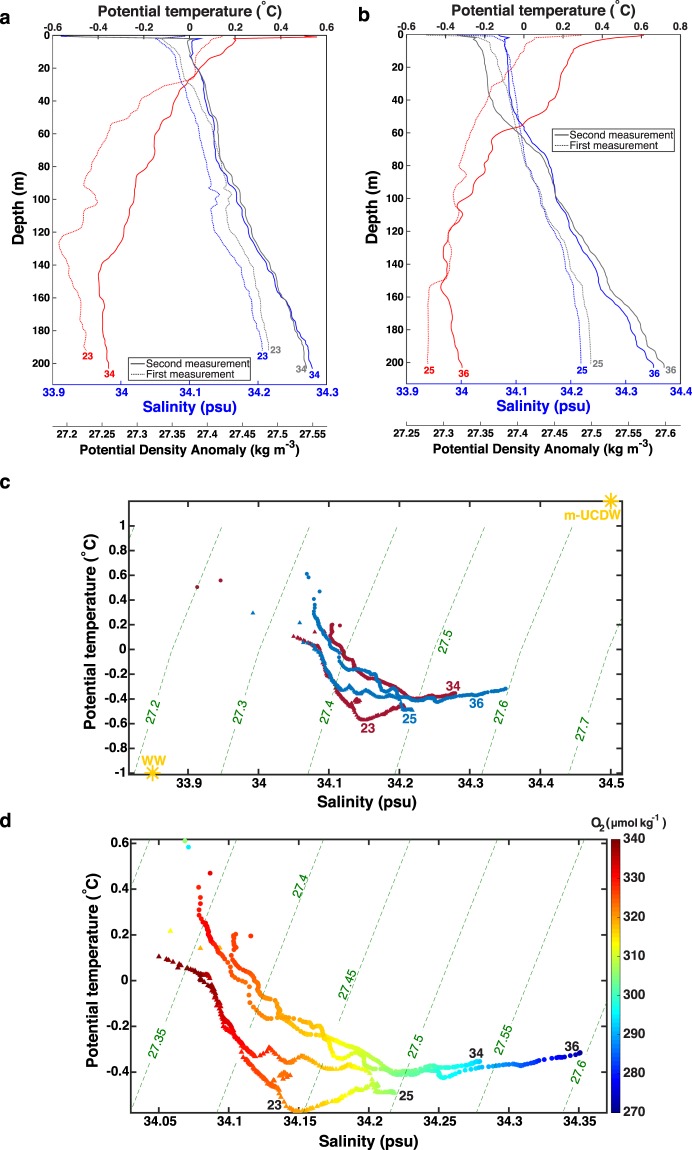
Profiles of potential temperature (°C, red line), salinity (PSU, blue line) and potential density anomaly (kg m^−3^, gray lines) from selected CTD casts sampled in Maxwell Bay in December 2017: **(a)** cast 23, repeated in cast 34, and **(b)** cast 25, repeated in cast 36. **(c)** Theta-S diagram with contours of potential density anomaly (kg m^−3^, green lines) from casts 23 and 25 (triangles), which were repeated 9 days after with casts 34 and 36 (circles). Reoccupied casts are shown with the same color. The water types corresponding to modified Upper Circumpolar Deep Water (m-UCDW) and Winter Water (WW) are included (yellow stars). **(d)** Zoomed-in Theta-S diagram with dissolved oxygen values (µmol kg^−1^, colors) for each cast.

The sill separating Maxwell Bay from the Bransfield Strait is sufficiently deep to permit intrusions of m-UCDW into Maxwell Bay in the form of relatively warm and salty subsurface eddies or filaments. Indeed, traces of episodic intrusions of m-UCDW were found close to the mouth of Maxwell Bay (cast 23, repeated in cast 34) and closer to its head (cast 25, repeated in cast 36) during the December 2017 campaign. Specifically, in cast 23 a thin lens of warmer and saltier water was found at 100 m depth, being part of a larger structure located between 90 and 125 m depth (Fig. 8a). In addition, there was a thicker tongue of warmer and saltier water centered at about 180 m depth in cast 23. This interleaving of intruding m-UCDW was not observed 9 days later in cast 34 (Fig. 8a), highlighting the transient nature of these events. Instead, we found that between 80 m and 200 m depth the water column warmed 0.16 °C, got 0.044 PSU saltier, 0.029 kg m^−3^ denser and 9.78 μmol kg^−1^ less oxygenated (on average) (Fig. 8a,c,d). The largest warming (up to 0.27 °C) was observed at 127 m depth, while the greatest salinity gain (up to 0.067 PSU), potential density gain (up to 0.051 kg m^−3^) and oxygen loss (up to 13.71 μmol kg^−1^) occurred at 194 m depth. Consequently, the oxygen loss, salinity gain and potential density increase are stronger in the deeper part of the water column sampled with these casts. This can be seen in the Theta-S diagram (Fig. 8c) as a displacement of the bottom part of casts 23 and 25 toward warmer, saltier and denser values in casts 34 and 36 respectively. Similarly, the oxygen loss is larger in the bottom part of the repeated casts (Fig. 8d). This is the result of the mixing with m-UCDW that followed the observed interleaving.

Similarly, cast 25 presents 3 lens of warmer and slightly saltier water centered at about 85, 110 and 140 m depth, which appear highly eroded 9 days later in cast 36 (Fig. 8b), after leading to an average warming (0.06 °C), salinity gain (0.057 PSU), oxygen loss (14.18 μmol kg^−1^) and potential density increase (0.044 kg m^−3^) between 80 and 200 m depth (Fig. 8b–d). Furthermore, the largest warming (up to 0.16 °C), salinity gain (up to 0.122 PSU), oxygen loss (up to 29.55 μmol kg^−1^) and potential density gain (up to 0.091 kg m^−3^) was observed at 200 m depth (Fig. 8b–d). The interleaving and mixing of m-UCDW with the waters inside Maxwell Bay can explain these changes. In contrast, the upper 60 m of these casts (25 and 36) are influenced by the meltwater accumulation induced by the flood tide toward the head of Maxwell Bay. Specifically, the upper 60 m are on average warmer (0.29 °C), fresher (0.002 PSU) and less dense (0.016 kg m^−3^) in cast 36 (flood tide) than in cast 25 (slack tide after) (Fig. 8b).

We also found a wide lens of warmer water between 100 and 140 m depth in cast 35 (located half way between casts 34 and 36, not shown). These results suggest that interleaving is an important and ubiquitous mechanism by which strongly modified UCDW enters Maxwell Bay. Therefore, the intrusions of m-UCDW and its mixing with the inner waters of Maxwell Bay results in an increase of the temperature, salinity and density and a decrease of the dissolved oxygen content of the subsurface waters inside Maxwell Bay.

## Summary and Conclusions

Two oceanographic campaigns accomplished in Maxwell Bay and tributary fjords (King George Island) in February 2016 and December 2017 allowed detecting changes in the hydrographic structure over tidal and intraseasonal timescales. We have also documented for the first time the entry of relatively warm subsurface waters (m-UCDW) into Maxwell Bay.

In the absence of strong winds and remote forcing, tides can play a significant role in changing the hydrographic structure in Maxwell Bay and their tributary fjords. The flood tide, strengthened by the funnel-shaped coastline, induces the accumulation of meltwater in the upper 70 m of the water column toward the head of Maxwell Bay, forming a layer with salinities below 34.11 PSU. Tidal straining and the internal tide may also play a role in the observed accumulation of low salinity water. Below 70 m, the flood tide can enhance the intrusion (and mixing) of m-UCDW into Maxwell Bay. An increased retention of meltwater during flood tide was also observed at the head of Marian Cove. In Collins Bay and Marian Cove (tributary fjords) the water column was found to be fresher and colder the closer to the ice-cliffs (located at their heads) with independence of the tidal phase.

Our hydrograhic data indicates a dominant residual estuarine circulation (two-layered) during the austral summer in Maxwell Bay, Marian Cove and Collins Bay. However, our measurements reached only the upper 200 m, so we cannot discard a three-layered residual flow in Maxwell Bay. Furthermore, tidal straining in strongly stratified fjords (like Marian Cove and Collins Bay) can partially oppose the estuarine circulation.

Winter convection is a commonly accepted mechanism for deep-water renewal in shallow-silled fjords^61^. Here we propose that the remnants of the Winter Water found at the bottom of Marian Cove are progressively eroded along the austral summer by tidal stirring and the breaking of the internal tide. This mechanism may be also at work in other Antarctic fjords with shallow submarine sills.

The advance of the austral summer is characterized by a buoyancy gain through warming and freshening of the surface layer influencing at least the upper 105 m in Maxwell Bay, the upper 80 m in Collins Bay, and the entire water column in Marian Cove. The magnitude of these changes is larger than the hydrographic variability due to the oscillating tides, the blowing winds and the fluctuations of the interior density field. Furthermore, the average variability in potential temperature (salinity) due to the tidal cycle only represents up to 25% (13%) of the variability due to the intraseasonal cycle of the austral summer in Maxwell Bay and Collins Bay. In Marian Cove however, tidal variability represents up to 11% of the intraseasonal variability in potential temperature and doubles the magnitude of the intraseasonal variability in salinity.

Finally, we found that m-UCDW occasionally intrudes into Maxwell Bay. The initial interleaving of m-UCDW with the waters inside the bay is followed by a substantial increase in temperature, salinity and density, and a decrease in dissolved oxygen throughout the depth range between 80 and 200 m. These intrusions have important implications for primary production (m-UCDW is rich in nutrients) as well as for the mass balance of the numerous tidewater glaciers bordering Maxwell Bay.

## Data and Methods

### Hydrographic stations

Hydrographic stations were sampled in February 2016 and December 2017. Measurements of water column temperature, salinity, depth, dissolved oxygen and turbidity were taken by using a SBE-19plus V2 CTD. The CTD casts were performed at the locations of Maxwell Bay and tributary inlets (Collins Bay, Edgell Bay and Marian Cove) indicated in Fig. 1b. In December 2017, the hydrographic measurements taken along the longitudinal axis of Marian Cove (Fig. 1b) were repeated 3 times (at flood tide, ebb tide and slack tide). CTD casts in Maxwell Bay and Collins Bay were not exactly repeated in space but they were sampled at close locations during flood, ebb and slack tide in December 2017. Due to logistical limitations, February 2016 casts were sampled down to approximately 100 m, while December 2017 casts were accomplished down to approximately 200 m depth. The dataset obtained was processed with the *Sea-Bird Data Processing 7.23.2* software following the typical data processing sequence suggested in the *Sea-Bird Data Processing* manual (page 20). The manufacturers declared accuracy of the CTD data is ±0.005 °C for temperature, ±0.0005 S/m for conductivity and ±2% of saturation value for dissolved oxygen (for Winter Water at −1.80 °C and 34.00 PSU its oxygen saturation value would be 359.69 μmol kg^−1^ while for m-UCDW at 0.5 °C and 34.5 PSU it would be 346.20 μmol kg^−1^).

The Hydrographic Service of the Chilean Navy provided the tidal information during the oceanographic samplings (http://www.shoa.cl/mareas/tablademarea.html). Reference station: Puerto Soberania. This station is located in the neighbouring Greenwich Island, less than 50 km away from Maxwell Bay and approximately on the same cotidal line for the principal tide constituent (M2)^75^.

### Meteorological data

Wind direction, wind intensity and surface air temperatures were obtained from a weather station installed in King George Island by the Chilean Navy (Capitanía de Puerto, Comandancia Marítima). A previous work found a very high correlation between the winds measured in the meteorological station in King George Island with the satellite wind data observed for the SSI Archipelago^57^, suggesting that ground-based meteorological data are representative of larger spatial scales.

### Estimation of the deepening of the isopycnal 27.4 caused by the barotropic tide amplification during flood tide in Maxwell Bay

Here we investigate the plausibility of a semidiurnal barotropic tide current of 40 cm/s causing the observed deepening of 20 m in the isopycnal 27.4 at the head sector of Maxwell Bay during flood tide.

A strengthening of the tidal current is expected inside Maxwell Bay when passing through its narrowest part (between the Barton Peninsula and Nelson Island, Fig. 1b), which communicates the mouth of Maxwell Bay with its head (where the meltwater retention has been observed at flood tide). The width of Maxwell Bay decreases from ~9 km at its mouth to ~6 km at this passage. By assuming the same average depth at the narrow passage and at the mouth of Maxwell Bay, and assuming that the volumetric tidal transport (Q) is conserved, we can estimate the tidal velocity at the narrow passage (v_2_) as: v_2_ = (A_1_/A_2_)*v_1_ = 9/6 * 0.4 m/s = 0.6 m/s. However, it is unlikely that this velocity will be maintained during the 6 h of the flood tide phase. Consequently, we use a more conservative estimate of the barotropic tidal velocity (40 cm/s) at this narrow passage. This is equivalent to considering a barotropic tidal velocity at the passage of 60 cm/s running only during 4 h of the 6 h corresponding to the flood tide. We now assume that only the water transported above the specific depth of the isopycnal 27.4 at the narrow passage (~40 m at cast 34, Fig. 2b) will contribute to deepen this isopynal as observed in the head sector of Maxwell Bay. Then, knowing the width of the narrow passage, the average velocity of the barotropic tidal current and the water depth of the isopycnal 27.4 at this passage, we are able to compute the volume of seawater (V) that the flood tide could drive across the narrow passage in the upper 40 m and into the head Maxwell Bay (V = 6000 m *40 m * (3600 s/h * 6 h * 0.4 m/s)). We assume that this volume of surface water (V) spreads uniformly across the head area of Maxwell Bay, and that the head area can be approximated by a rectangular area (A) of 12 km width and 9 km length. Then, we have that V/A should give us an estimate of the deepening (h) of the isopycnal 27.4 in the head of Maxwell Bay that could be caused by the incoming barotropic tide during flood tide. We finally obtain a deepening of 19.2 m of the isopycnal 27.4. Consequently, we can conclude that, given the funneling effect of the coastline inside Maxwell Bay, a barotropic tidal current of 40 cm/s could cause the observed deepening of the 27.4 isopycnal in the head of Maxwell Bay.

## Data Availability

The datasets generated during the oceanographic research cruises and/or analyzed during the current study are available from the corresponding author on request.
